# Assessing the reach and engagement with the ‘*How To Save A Life*’ mass media campaign on drug-related death prevention in Scotland

**DOI:** 10.1080/09687637.2023.2262735

**Published:** 2023-09-27

**Authors:** M. Anderson, A. M. Atkinson, A. McAuley, H. R. Sumnall, M. E. Glancy, H. A. Bloomfield, K. M. A. Trayner

**Affiliations:** a MRC/CSO Social and Public Health Sciences Unit, University of Glasgow, Glasgow, UK; b Scottish Drugs Forum, Glasgow, UK; c Public Health Institute, Liverpool John Moores University, Liverpool, UK; d School of Health and Life Sciences, Glasgow Caledonian University, Glasgow, UK; e Public Health Scotland, Glasgow, UK

**Keywords:** Naloxone, mass media campaigns, reach, engagement, harm reduction, public health

## Abstract

‘*How To Save A Life’* (HTSAL) was a mass media campaign on drug-related death prevention which ran in Scotland from August 2021 to January 2022. It aimed to increase awareness of how to respond to an opioid overdose, and the uptake of take-home naloxone (THN). The objective of this study was to determine the reach and engagement with the campaign. Methods included a descriptive analysis of data from media sources, the campaign website, and an online naloxone training course. A quantitative content analysis was conducted on media articles.

The campaign generated 57,402,850 non-unique impressions (the total number of times the campaign was seen or heard), and unique reach (the number of people who were exposed to the campaign) figures of 2,621,450. Engagement with the campaign was positive, and 96% of print/digital media articles had a positive view of the campaign. There were 40,714 visits to the campaign website, leading to 8,107 clicks to the free naloxone training course, and 3,141 clicks to order a free naloxone kit.

This study showed that mass media campaigns on drug policy topics can achieve high levels of reach and engagement. There was a clear progression from viewing campaign materials, to visiting the campaign website, to completing naloxone training. Our research suggests that mass media campaigns can be used to disseminate harm reduction messages to the general public.

## Introduction

Scotland is currently experiencing record levels of drug-related deaths, mainly overdoses involving opioids and benzodiazepines. In 2021, there were 1330 drug related deaths (DRDs), an estimated rate of 22.5 per 100,000. This translates to among the highest recorded rates in Europe and 3.7 times than the rate of the UK as a whole (National Records of Scotland, 2022). Drug-related deaths are associated with factors including socioeconomic deprivation, polydrug use, low rates of retention in opioid agonist treatment (OAT), and reduced treatment funding (Kalk et al., 2018; McPhee et al., 2020; Parkinson et al., 2018; Tweed et al., 2022).

Reducing and addressing drug-related mortality in Scotland is paramount, and a crucial policy objective for the Scottish Government. A ‘national mission’ to reduce drug deaths has identified a range of measures to prevent DRDs, including improved access to OAT, safer consumption sites, and distribution of take-home naloxone (THN) (Scottish Government, 2021). THN programmes are a fundamental component of the national mission; naloxone, when administered immediately after an opioid overdose has occurred, reverses the effects of opioids and significantly reduces the likelihood of mortality (McDonald & Strang, 2016). Due to the potentially brief window between overdose and death, it is important that naloxone can be administered quickly by people present at the scene of an overdose.

In 2011, Scotland was the first country in the world to introduce a national naloxone programme (McAuley et al., 2012). Research has shown that this policy reduced the proportion of fatal opioid overdoses that occurred within four weeks of leaving prison, a time of particularly heightened risk (Bird et al., 2016). By the end of 2021, 94,170 naloxone kits had been supplied, through drug treatment services, homelessness services, mental health services, community pharmacies, prisons, community prescribing, and the Scottish Ambulance Service (SAS) (Public Health Scotland, 2022). In 2020-21, 1,377 of the 6,447 THN kits distributed as repeat supplies from community outlets, prisons, and the Scottish Ambulance Service (SAS) were due to the previous kit being reported as having been used to treat an opioid overdose (Public Health Scotland, 2022).

THN programmes involve a training session on how to recognise an overdose, administer naloxone, and provide first aid (Clark et al., 2014). Naloxone training is important for increasing the effectiveness of community THN programmes, improving factors such as confidence to administer naloxone. A review of systematic reviews by Razaghizad et al. (2021) found that overdose education and naloxone distribution (OEND) programmes are effective in producing long-term knowledge about opioid overdose, improving attitudes towards naloxone, preparing participants to safely and effectively manage overdoses, and can effectively reduce opioid-related mortality. A brief overdose education is sufficient for naloxone distribution to people who use opioids, having been shown to significantly increase comfort with overdose recognition, response, and naloxone administration among first time naloxone recipients (Behar et al., 2015). Furthermore, a study of people who use opioids in Baltimore, USA, found that insufficient overdose training was associated with decreased access to THN (Dayton et al., 2019). A study of support groups for family members of opioid users in Massachusetts, USA, found that most attendees had received overdose education and naloxone training, suggesting these are important components of supporting people who use opioids (Bagley et al., 2015).

**Figure 1.**
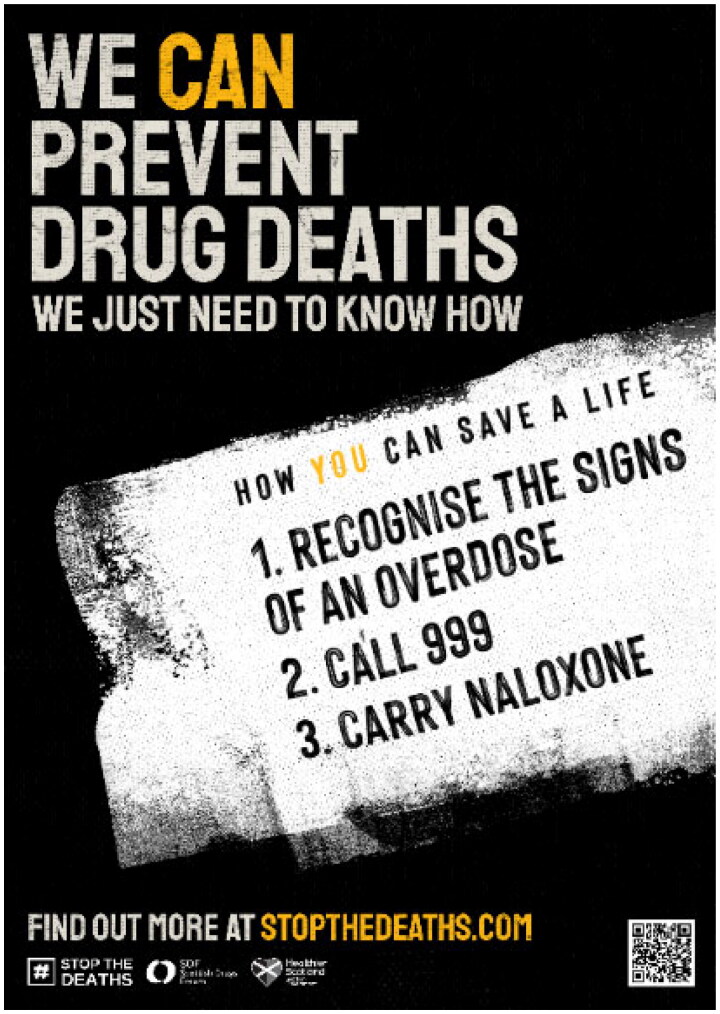
“How to save a life” mass media campaign poster.

The ‘*How To Save A Life*’ (HTSAL) campaign was a nationwide mass media campaign to raise awareness of the signs and symptoms of an overdose and encourage the public to carry THN (Trayner et al., 2022). The campaign was commissioned by the Scottish Government and delivered by Scottish Drugs Forum (SDF), a non-government organisation specialising in drug policy. It ran from 30th August 2021 to 24th October 2021, followed by an additional booster campaign from 13th December 2021 to 13th January 2022 (Appendix A). The campaign was delivered nationally using billboards in transport hubs, shopping centres, and outdoor locations, and adverts on TV, radio, and social media. It also featured the voice of a prominent Scottish television and film actor. The campaign materials (Figure 1, Appendix B) directed people to the Stop The Deaths website (https://www.stopthedeaths.com/), which provided further information about how to recognise an overdose. The website also provided two external links, one to a free naloxone and overdose preventing eLearning course offered by SDF, another to a naloxone ‘click and deliver’ service provided by Scottish Families Affected By Alcohol & Drugs (SFAD), which allowed people to order a THN kit online directly to their home in Scotland. The two primary objectives of the campaign were 1) to increase awareness of drug deaths, the signs and symptoms of an overdose, and how to respond to an overdose, and 2) increase the supply of THN. The HTSAL campaign was the most wide-ranging drug-death prevention campaign ever conducted in Scotland.

Mass media campaigns use coordinated communications delivered across multiple media platforms to deliver public health messages at the population level, with the aim of increasing knowledge, influencing attitudes, and/or motivating behaviour change (Wakefield et al., 2010). There is evidence that these forms of campaign can increase knowledge and awareness of public health issues. However, there is mixed evidence about their effect on behaviour change, a tendency for any observed behavioural effects to be short-term, and there is little evidence that campaigns are effective in reducing illicit substance use (Stead et al., 2019).

Evaluation of mass media campaigns tend to include common metrics such as impressions, reach, and engagement to assess the scale and success of the campaigns. Impressions are a common indicator of target audience reach using in assessment of online marketing activity, and are considered an important metric of campaign success, particularly when the aim is to increase awareness of a topic and reach a new audience (Teoh et al., 2020). It is usual for campaigns using public billboards, TV/radio, and social media advert to report tens to hundreds of millions of impressions (defined as the non-unique number of times materials were seen overall). For example, a campaign to reduce sugar consumption in Los Angeles generated 359 million billboard impressions and 82 million video impressions (Barragan et al., 2014). Another sugar reduction campaign in rural southern USA using TV, radio, and social media gained approximately 19 million video impressions, 2 million audio impressions, and 5 million impressions from social media and internet radio (Farley et al., 2017). A 10-week social media campaign to reduce human papillomavirus (HPV) vaccine misinformation in Louisiana USA gained 370,000 impressions, with a unique reach of 33,000 individuals (Sundstrom et al., 2021). In relation to COVID-19, an evaluation of a campaign to improve vaccine confidence found that campaign impressions were positively associated with vaccine uptake (Williams et al., 2023). Digital media campaigns for other health topics, such as healthy eating, continued to be successful in generating impressions throughout the pandemic (Grantham et al., 2021). However, it is not possible to make a direct comparison with COVID-19 campaigns, as this was an international health issue affecting the whole population, and HTSAL concerned a relatively small and stigmatised section of the population.

Due to the ability to track activity across multiple sites, digital and social media campaigns tend to produce more detailed analytics than broadcast and billboard campaigns. As well as impressions, digital and social media are typically evaluated on metrics such as unique reach (the number of unique individuals who have seen campaign materials), the proportion of viewers who click through to campaign websites, engagement in terms of likes and shares, and the sentiment of comments left by users in response to materials. Social media comments can be thematically analysed to understand the tone and sentiment of public engagement, providing insight into the impact of a campaign on public discussion (Schlichthorst et al., 2019). For example, in their evaluation of a social media campaign designed to promote health seeking attitudes for depression, Hui et al. (2015) found that it was possible to convert millions of impressions into thousands of active clicks at a reasonably low cost. Social media is also particularly useful for rapid diffusion of materials to specific target audiences, especially younger audiences and others who may not be reached by traditional media (Jawad et al., 2015). High levels of user engagement are associated with positive emotional messaging, the delivery of factual information, the inclusion of celebrities in campaign materials, and the use of multimedia (e.g., combining text posts, images, and videos) (Card et al., 2018).

This study was part of a wider multi-method evaluation of the HTSAL campaign (Trayner et al., 2022). Other studies focused on assessing the impact of the campaign on the community supply of THN in Scotland using a segmented time-series analysis, which found that the campaign had a large and significant impact on supplies for the duration of the campaign (Trayner et al., 2023). Another strand used a panel survey to examine awareness and recall of the campaign among the general public, which found a high awareness of the campaign among the general public and that exposure to campaign materials was associated with an increased knowledge of overdose signs and symptoms (Sumnall et al., 2023). Conversely, this study focuses on analyses of media data sources and website analytics, examining the reach and engagement with the HTSAL campaign. We had three objectives: 1) examine the reach of the campaign, defined as how many people were exposed to campaign materials, 2) assess and quantify public engagement with the campaign, both on social media and in national and local print/digital media coverage, and 3) quantify the effect of the campaign on further action, including visiting the campaign website, and registering for and completing naloxone training.

## Methods

### Design

We combined and triangulated several data sources. A summary of each data source and key outcomes can be viewed in Table 1. The study period was 30th August 2021 to 16th January 2022, which included both the main and booster campaign. Ethical approved was not required as this analysis involved qualitative analysis of media articles, and secondary analysis of data that did not relate to individuals.

**Table 1.** Summary of data sources and methods used in the media evaluation of the “how to save a life” mass media campaign.

Data source	Methods	Key outcomes
***Media campaign data***	Descriptive analysis of data provided by media providers (TV, radio, outdoor billboards, transport hubs) and social media (Facebook/Instagram) Time period: 30^th^ August 2021– 13^th^ January 2022 Time period (social media): 20^th^ September 2021 – 13^th^ January 2022	***Reach:*** number of individuals who saw the HTSAL campaign ***Impressions:*** total number of times HTSAL material were seen/heard
***Social media comments***	Quantitative content analysis of comments left in response to the social media adverts, which showed the campaign video advert and a link to stopthedeaths.com. Time period: 20^th^ September 2021 – 13^th^ January 2022	Thematic codes derived from quantitative content analysis
National and local ***print and digital media articles***	Quantitative content analysis of articles that referred to the HTSAL campaign. Time period: 1st August 2021- 18th January 2022	Support of the campaign (positive, negative, neutral) Thematic codes derived from quantitative content analysis
Scottish Drugs Forum (SDF) ***Stop the Deaths website analytics and naloxone eLearning statistics***	Descriptive analysis of website analytics and naloxone eLearning statistics Time period (website): 30^th^ August 2021 – 14^th^ January 2022 Time period (eLearning): 30^th^ August 2021 – 16^th^ January 2022	Number of website visits Link clicks to naloxone eLearning Link clicks to order naloxone from SFAD Number of people who registered for naloxone eLearning Number of people who completed naloxone eLearning

### Analysis and outcomes

#### Reach and impressions

To assess campaign reach, we conducted a descriptive analysis of data supplied by the media providers (advertising purchasers, social media sites) who facilitated the campaign on TV, radio, billboards, transport, and social media. Media data were reported from 30th August 2021 to 13th January 2022. The main metric available for all sources were ‘impressions’, defined as the number of times campaign materials were estimated to have been seen or heard. For digital TV, digital radio, and social media, ‘reach’ data was also reported which includes the number of unique individuals who saw or heard the materials. Reach figures for separate broadcasters could not be combined, nor could reach figures from the main and booster campaigns, as there is likely to be overlap in viewership. The main radio campaign was delivered from 30^th^ August 2021 to 24^th^ October 2021, via broadcast and digital radio. Analytics included metrics for adverts targeted at digital listeners through desktop applications, smart speakers, and mobile phones.

### Social media

The social media campaign promoted the HTSAL video advert on Facebook and Instagram. A longer version of the campaign video was used on social media, which was not possible on the TV advert. The social media campaign was presented to platform users in the whole of Scotland, and then targeted to areas not specifically covered by the radio campaign, and postcode areas identified through the Scottish Index of Multiple Deprivation (SIMD) as the most deprived 20% in the country (Ralston et al., 2014).

Social media data included variables such as link clicks (to the campaign website), cost per click (CPC), click through rate (CTR), post engagement, and 3-second video plays. These data were used to assess the extent to which the campaign reached its intended audience and in prompting further action.

Engagement with the campaign by members of the public was assessed by conducting a quantitative content analysis (Atkinson et al., 2019) of comments made in response to campaign materials on social media. This involved a thematic coding, and descriptive statistics calculating the proportion of comments that referred to each theme. The comments available for analysis did not include comments that had been deleted by social media administrators.

### Print/digital media

The ProQuest Factiva^TM^ database was used to identify articles in all UK countries (i.e., England, Scotland, Wales, and Northern Ireland) that referenced the campaign between 1^st^ August 2021 - 18^th^ January 2022. Representation of the campaign in print and digital media articles was undertaken using quantitative content analysis of newspaper articles to identify common themes that arose across articles (Atkinson et al., 2019). Articles included those with the campaign as the main focus, where all content focused on the campaign, and those about a broader issue such as DRDs, which also mentioned the campaign.

### Campaign website and eLearning course

We used website analytics from the campaign website and naloxone eLearning training course platform to assess further engagement with the campaign. Website analytics were obtained from 30th August 2021 to 14th January 2022. This included data on traffic, page views, and button clicks. Of particular interest were the ‘free eLearning’ and ‘get naloxone’ buttons. These button clicks were important outcomes for the campaign, as each click represented a willingness to seek further information. The SDF naloxone eLearning registrations and completions were measured from 30th August 2021 to 16th January 2022. Analysis involved comparing the absolute and proportionate increases to the baseline pre-campaign figures.

## Results

### Reach, impressions, engagement, and costs of the media campaign

Delivery through TV, rural radio, transport hubs, and billboards generated 53,171,977 impressions, at a total cost of £395,045 relating to both the main campaign and booster. Scottish Television (STV, a national public broadcast channel) reached 2,621,450 individuals, with another 1,510,058 reached by Sky Adsmart (Sky TV adverts which can be targeted to specific viewer demographics). Digital radio delivered 1,509,447 impressions, with a reach of 257,323 unique listeners (Table 2).

**Table 2.** “How to save a life” mass media campaign metrics and costs.

Media	Impressions^a^	Reach^b^	Spend
TV broadcast	25,633,983	N/A*	£162,011
STV National	22,282,325	2,621,450	£100,000
SKY Adsmart	3,276,000	1,500,000	£38,020
SKY Adsmart Targeted (Inverclyde)	75,658	10,058	£3,026
ITV Borders	N/A**	N/A**	£20,965
Outdoor (billboards, etc.)	11,499,083	N/A	£97,188
Transport	4,955,045	N/A	£86,741
Rural radio	108,000	108,000	£10,050
Digital radio^c^	1,509,447	257,323	
Misc.	981,406	N/A	£6,714
TV broadcast booster (STV National)	7,069,920	1,767,480	£36,000
Outdoor booster	2,924,540	N/A	£10,171
Total (main campaign and booster)	54,681,424	N/A*	£395,045
Main campaign	44,686,964	N/A*	£348,874***
Booster campaign	9,994,460	N/A*	£46,171

^a^ Impressions: defined as the number of times seen or heard (TV, radio, outdoor and transport).

^b^ Reach: defined as the number of unique individuals who saw or heard the campaign (TV and radio only).

^c^ Facilitated by a separate media provider, analytics report did not include spend.

* Cannot combine unique reach figures from different broadcasters.

** The media report did not include impressions/reach data for ITV borders.

*** The main campaign was discounted from £362,704 to £348,874.

There were 57 advertising slots purchased on social media, for a total cost of £10,000. The adverts generated 2,721,426 impressions, 627,449 post engagements, 588,511 3-second video plays, and a unique reach of 483,592 people. In total, the advert generated 35,878 link clicks to the campaign website, amounting to a cost-per-click (CPC) of £0.28. The click-through rate (CTR), i.e., the proportion of impressions that led to a click, was 1.3%. The unique CTR, i.e., the proportion of unique reach that led to a unique click, was 3.8% (Table 3).

**Table 3.** “How to save a life” social media impressions, reach, and engagement.

Measure	Count
Number of advertising slots purchased	57
Total spend	£10,000
Impressions	2,721,426
All of Scotland	1,251,905
Deprived areas*	1,140,123
Areas outside of main radio coverage	329,398
Post engagement	627,449
3-second video plays	588,511
Reach**	483,592
All of Scotland	308,113
Deprived areas	259,523
Areas outside of main radio coverage	74,995
Link Clicks to the Stop The Deaths website	35,878
All of Scotland	18,140
Deprived areas	14,786
Areas outside of main radio coverage	2,952
Comments	897
Engagement rate	32%
Unique click-through rate	3.78%
Click-through rate	1.32%
Cost-per-click	£0.28
All of Scotland	£0.28
Deprived areas	£0.27
Areas outside of main radio coverage	£0.34

^*^ Using an export of postcodes from the Scottish Index of Multiple Deprivation, we targeted the lowest 20% based on income, crime, education etc.

** This figure represents genuine unique reach. If an individual is shown the advert twice, e.g., once as part of ‘all of Scotland’ targeting and once as part of ‘deprived areas’ targeting, the duplicate has been removed. Consequently, the total figure is lower than the combined sub totals.

Public sentiment towards the campaign was assessed by analysing social media comments (Table 4). A total of 730 comments were available for analysis. Nine key themes were identified, the most common of which was debating political responsibility for Scotland’s high rate of DRDs (27%, n = 198). Comments also commonly discussed solutions to DRDs other than naloxone, such as stating that drugs should be legalised or that more treatment options were required (23%, n = 166). A minority of comments (8%, n = 56) contained derogatory and stigmatising comments towards people who use drugs.

**Table 4.** “How to save a life” mass media campaign thematic analysis of social media comments.

Comment sentiment	Proportion of comments
Debating political responsibility for the issue (e.g., Scottish or UK government)	198 (27%)
Discussion of solutions other than naloxone (e.g., legalisation, treatment and rehabilitation law enforcement)	166 (23%)
Discussion of causes or consequences of drug use (e.g., trauma, lack of purpose)	123 (17%)
Empathy towards people affected by the issue	77 (11%)
Statement of having been personally affected by the issue (e.g., personal/family lived experience)	70 (10%)
Derogatory remarks towards people who use drugs (e.g., ‘personal choice’, stigma, don’t deserve help)	56 (8%)
Positive sentiment towards the campaign (e.g., praising campaign directly, encouraging people to do the training)	43 (6%)
Negative sentiment towards the campaign (e.g., this should be a government responsibility not members of public)	24 (3%)
Mixed sentiment towards the campaign (e.g., stating the campaign was good but doesn’t do enough to address root causes)	9 (1%)

We identified 28 articles that referred to the HTSAL campaign, that were included in the media sentiment analysis (Table 5). With regards to overall sentiment, 96% (n = 27) reported positively on the campaign. Approximately two-thirds (61%, n = 17) included the HTSAL campaign as the main focus of the article and half (50%, n = 14) had DRDs as the main focus.

**Table 5.** “How to save a life” mass media campaign thematic codes identified through content analysis of print and digital media articles that referenced the campaign.

Thematic codes identified through content analysis	Proportion of articles, N (% of N)
**Overall sentiment**	
Positive	27 (96%)
Neutral	1 (4%)
Negative	0 (0%)
**Main focus of article**	
The HTSAL campaign	17 (61%)
Drug-related deaths	14 (50%)
Martin Compston support/voiceover	7 (25%)
**Themes present in article** *	
Drug-related deaths	27 (96%)
Stop the Deaths	24 (86%)
Naloxone	22 (79%)
Key campaign aims of raising awareness of signs of overdose and naloxone availability	22 (79%)
Stop The Deaths website	17 (61%)
Public ‘intervention’ encouraged, by carrying naloxone and intervening in overdoses	15 (54%)
Used the campaign slogan, ‘Save a Life’ or ‘Save Lives’	12 (43%)

* Multiple themes could be present in each article.

With regards to a quantitative content analysis of the themes present in each article, the most common theme was references to DRDs in Scotland (96%, n = 27). Some key campaign messages were reported more frequently than others, including ‘Stop the Deaths’ (86%, n = 24) (the main phrase used by the HTSAL campaign) and reporting the key campaign aims (79%, n = 22).

Campaign materials encouraged people to visit the website, where visitors were provided further links to a naloxone eLearning course, and a naloxone click and deliver service. From 30^th^ August 2021 – 14^th^ January 2022, the campaign website received 40,714 visits, from 34,321 unique visitors. Relating to the source of website visits, 39% came from internet search engines, and 32% were direct visits to the URL, which was promoted in the campaign messaging. Just under one-quarter (23%) came from direct link clicks on social media. Although the proportion of visitors who left the website after viewing one page (the ‘bounce rate’) was high (88%), key campaign messaging and links were provided on the home page, the first page seen. There were 8,107 clicks of the ‘eLearning’ button, and 3,141 clicks of the ‘get naloxone’ button (Table 6).

**Table 6.** “How to save a life” mass media campaign website analytics.

Measure	Count
Page views	47,735
Total visits	40,714
Visits from a search engine	15,906 (39%)
Visits directly to URL	13,120 (32%)
Visits from social media	9,287* (23%)
Visits referred through other websites	2,192 (5%)
Unique visitors	34,321
Bounce rate (leave after viewing one page)	88%
‘eLearning’ button clicks	8,107
‘Get naloxone’ button clicks	3,141

* Social media analytics showed 35,878 clicks to the website, but website analytics show 9,287 visits. This may be caused by 1) a natural drop off from people clicking the link but not allowing the page to load, and 2) website host analytics not accurately tracking data from social media traffic. The real number of visits from social media is likely closer to 35,878 than 9,287.

Before the campaign, the eLearning had been live for approximately three years, accumulating 5,942 registrations and 4,610 completions, a 78% completion rate. From 30th August 2021 to 16th January 2022, there were an additional 4,318 registrations and 2,876 completions, a completion rate of 67%. As a cumulative total, the campaign increased total registrations to 10,260 (a 73% increase) and total completions to 7,486 (a 62% increase) (Table 7).

**Table 7.** “How to save a life” mass media campaign naloxone and overdose prevention eLearning statistics.

	Registered	Completed	Completion rate
**Pre-campaign (28^th^ August 2018 – 29^th^ August 2021))**	5,942	4,610	78%
**During Campaign (30^th^ August 2021 – 16^th^ January 2022)**	4,318 (73% increase*)	2,876 (62% increase*)	67%
**Total after campaign**	10,260	7,486	73%

* Relative to pre-campaign.

## Discussion

We undertook an analysis of the media reach, coverage, and engagement with the HTSAL campaign, which was the most wide reaching and first national campaign on drug policy delivered in Scotland, which aimed to increase awareness of drug-related deaths and the uptake of take-home naloxone. This work was a component of a wider evaluation of the campaign, with other studies focusing on national distribution of THN (Trayner et al., 2023) and public awareness of the campaign (Sumnall et al., 2023).

It was not possible to make comparison to campaigns more topically similar to HTSAL, such as the Release *National Overdose Awareness and Naloxone* billboard campaign, which launched four months before HTSAL (Bernard & Garius, 2021). This was not on the same multimedia scale as HTSAL, and there are no published evaluations wherein metrics may be compared. A campaign in the USA by the Centres for Disease Control and Prevention (CDC) did use a multimedia approach to increase public awareness of the dangers of non-prescription opioid use, generating 141,281,311 impressions and 360,294 clicks to an overdose information page on their website (CDC, 2017). However, this was not independently evaluated and was critiqued for using negative messaging and a failure to offer any solutions for people affected by the issue (Diep, 2017). Therefore, it is more useful to compare HTSAL to wider range of health-related mass media campaigns. Our studies found that the HTSAL campaign had a wide reach. In particular, the billboard and broadcast elements of the campaign generated an estimated 54.7 million non-unique impressions. These figures are consistent with other mass media campaigns, which tend to generate tens to hundreds of millions of impressions in similar timeframes (Barragan et al., 2014; Farley et al., 2017). Although it is difficult to make direct comparison due to geographic population differences and variation in campaign scale, the impressions generated by HTSAL were not as high a campaign to reduce sugar consumption in Los Angeles, which generated 359 million billboard impressions in an initial eight week run, and 82 million video impressions from a twelve-week run on television (Barragan et al., 2014). However, it was comparable to another sugar reduction campaign in rural southern USA, which used TV, radio, and social media to gain approximately 19 million video impressions, 2 million audio impressions, and 5 million impressions from social media and internet radio (Farley et al., 2017). In comparison, HTSAL generated over 14 million billboard impressions and over 32 million TV impressions. The social media campaign generated another 2.7 million impressions. Again, this was comparable to other social campaigns, generating fewer impressions than billboard/broadcast, but more precisely targeted (Hui et al., 2015; Jawad et al., 2015). The social media campaign performed favourably in comparison to a 10-week HPV vaccine misinformation campaign in Louisiana, which generated 370,000 impressions (Sundstrom et al., 2021).

The unique reach (the number of people who saw the campaign) indicated that the main STV campaign reached 2.6 million people, the Sky Adsmart campaign reached 1.5 million, the digital radio campaign reached over 250,000 people, and the social media campaign reached over 480,000 people. These figures suggest that the HTSAL campaign was seen by a high proportion of the Scottish population (5.46 million in 2020), with the main STV campaign alone potentially reaching almost half the population. These findings can be triangulated with reference to other strands of this evaluation, in which a nationally representative panel survey found that 31% of the Scottish population recognised the campaign unprompted, rising to 61% with prompting (Sumnall et al., 2023).

Engagement with the campaign was positive. The campaign was well represented in print and digital media, 96% of articles reporting positively on the campaign. For comparison, Atkinson et al. (2019) analysis of media representations of proposals to introduce drug consumption rooms (DCRs) identified 174 articles, over a two-year period, of which 67% were positive and 20% were negative. The HTSAL campaign messaging was prominent in most coverage (79%) and the campaign itself was the main focus of the majority of articles (61%). Positive representations in a variety of media outlets with diverse political stances could suggest the HTSAL campaign was supported across the political spectrum, which is important as lack of cross-party support for certain interventions, such as DCRs, has been a barrier in preventing the most effective responses to drug-related deaths (Atkinson et al., 2019; Nicholls et al., 2022). The success of the campaign demonstrated that it is possible to achieve positive media coverage and successful metrics in a geographical context where substance use treatment can be politically divisive (Atkinson et al., 2019). The combined use of social media and a campaign website may be the most cost-effective way to generate traffic to further source of information, particularly if there are geographic barriers that would limit billboard and broadcast campaigns.

There was substantial engagement with the social media campaign, in terms of likes, shares, video plays, link clicks, and comments. Therefore, it would appear the use of multimedia, messaging that was both emotional and factual, and a high-profile celebrity voiceover contributed to engagement (Card et al., 2018). However, not all engagement on social media was positive or relevant to the campaign objectives. Quantitative content analysis of social media comments indicated the most common response was to debate political responsibility for drug deaths in Scotland, rather than responding directly to the campaign messaging. Furthermore, a minority of comments contained stigmatising or derogatory language towards people who use drugs. Similarly, Schlichthorst et al. (2019) evaluation of a masculinity and suicide campaign also found an overall positive response with a minority of negative comments. However, they also found that comment themes were more closely linked to the campaign messages (e.g., the need to express emotions), whereas the HTSAL campaign appeared to spark ongoing political debates that were not the focus of the campaign. Despite this, comment engagement was very low in comparison to video views and link clicks to the campaign website, indicating substantial engagement with the materials by people who did not comment on posts. Furthermore, other stands of this evaluation also demonstrated that exposure to campaign materials was associated with harm reduction knowledge, an increase in the signs and symptoms of an overdose, and that there is a high support for harm reduction policies amongst the general public (Sumnall et al., 2023). We have found that harm reduction is an appropriate topic for general public, and therefore momentum could be built on this finding to continue to increase support for drug policy and harm reduction interventions (Sumnall et al., 2023). That the campaign topic was drug-related deaths indicates that mass media campaigns may be suitable for similar harm-reduction topics, for example, the availability of new antiviral drugs to treat blood-borne viruses such as the hepatitis C virus (HCV).

There was a demonstratable progression from exposure to campaign materials to further information seeking and action. Exposure to campaign materials created substantial traffic to the Stop The Deaths main campaign website, which received 34,321 unique visitors. Although the reach from social media (483,592 people) was relatively small in comparison to the millions reached through the TV, radio, and billboard elements, the proportion of social media users who clicked a link to the Stop The Deaths website demonstrated a clear pathway from viewing the advert to further information seeking. Social media clicks accounted for approximately one-quarter (23%) of all website traffic, despite social media impressions (2.7 million) being approximately only one-twentieth (5%) of the TV/billboard impressions (54.7 million). This suggests social media impressions led to a higher level of website traffic, likely because it is easier to click an embedded link on social media than recall the campaign message from an advert and actively search for it. Social media was also less expensive than advertising on other platforms (£10,000 compared to approx. £400,000 for TV/billboards etc.). Social media achieved a cost-per-click of £0.28, which suggests a successful advertising campaign. Sundstrom et al. (2021) report that the national CPC average for health campaigns in the USA was $1.3 USD, equivalent to approximately £1.13 sterling (at time of writing). The click-through rate of 1.3% was notably very high compared to other social media campaigns, which are often less than one percent (Hui et al., 2015; Sundstrom et al., 2021). For example, in their evaluation of a social media campaign designed to promote help-seeking attitudes for depression, Hui et al. (2015) were able to convert approximately 12 million impressions into nearly 3,000 clicks to further information, a click-through rate of 0.02%.

**Figure 2.**
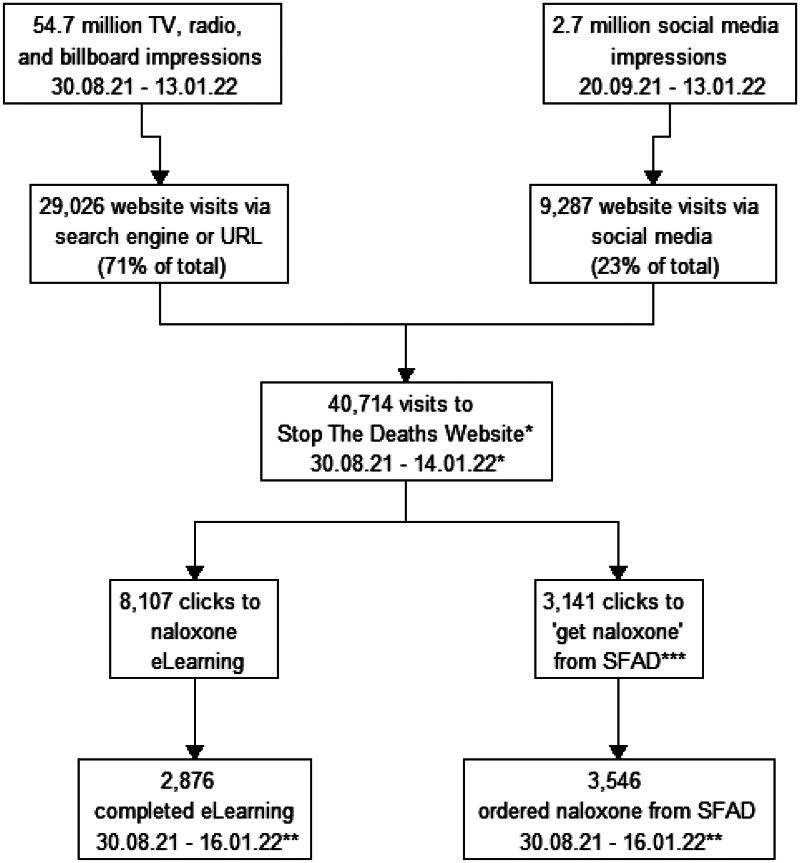
Flow chart illustrating exposure to “How to save a life” campaign materials and specific campaign related actions. *Website analytics recorded to the day after the campaign ended, 14th Jan 2023. **eLearning and SFAD data were reported weekly, therefore recorded to the end of the final week the campaign was active, 16th Jan 2022. ***The number of people who ordered naloxone is higher than the number who clicked ‘get naloxone’ on the campaign website. This is because people may have accessed the SFAD service via other routes.

There was progression from seeing the materials, to visiting the campaign website, to taking further action, such as completing the SDF naloxone eLearning and clicking the button to order a THN kit. Significantly, the campaign coincided with almost as many registrations for the naloxone eLearning as the previous three years since the eLearning was first launched and increased the number of eLearning completions by over half. This suggests the campaign could have had an impact on the uptake of an intervention, which evidence from systematic reviews indicates that not all campaigns successfully achieve (Stead et al., 2019) (Figure 2). This is important, as we know that naloxone training increases confidence to administer naloxone and respond to an overdose (Behar et al., 2015; Razaghizad et al., 2021). Furthermore, the campaign generated 3,141 clicks to ‘get naloxone’, taking people to the SFAD ‘click and deliver’ service, likely contributing to the 3,546 THN kits distributed by SFAD during the campaign period (Trayner et al., 2023). The strand of the evaluation which examined the impact of the campaign on the national community supply of THN found that the campaign contributed to a significant increase in naloxone distribution, with the majority of kits being issued as first supplies (i.e., people who had previously never accessed THN) and also to members of the public (Trayner et al., 2023). We know that communities directly affected by opioid use are more likely to engage in naloxone education (Bagley et al., 2015), and other stands of this evaluation also demonstrated that people who had witnessed an overdose previously were more likely to engage in follow up action (Sumnall et al., 2023).

### Strengths and limitations

This overall evaluation triangulated multiple data sources and methods, with research being conducted concurrently, so that different evaluation strands could be used to informed each other. This media strand of the evaluation drew together numerous data sources, including media analytics, published media articles, and analytics from the campaign website and eLearning course. This strand of the campaign used a primarily descriptive analysis, therefore has not provided any comparison of campaign reach amongst different demographic groups. However, this allowed us to broadly assess the media impact of the campaign. It should be noted that the information generated on impressions and reach was generated from the media advertising companies, and therefore could be subject to bias. Website analytics showed fewer visits referred via social media than indicated by social media analytics, likely due to website analytics underreporting social media traffic. Slightly different versions of the advert were used on TV and social media, which should also be acknowledged when comparing their relative analytics.

## Conclusion

The HTSAL mass media campaign was the widest ranging mass media campaign on drugs ever conducted in Scotland. This study showed that mass media campaigns on drug policy topics can achieve high levels of reach and engagement, with our results suggesting the campaign reached a substantial proportion of the Scottish population. HTSAL generated positive public engagement in print, digital and social media. The advertising campaign delivered on TV was the most effective in generating large numbers of impressions. The social media campaign was very effective at facilitating action after exposure, namely visits to the stop the deaths campaign website and registrations for SDF naloxone eLearning. This study demonstrated that mass media campaigns could prioritise TV, for wide population reach, and social media, for targeted reach and direct referral to further resources. Furthermore, this study also suggests that mass media campaigns can be used to disseminate harm reduction messages to the general public.

## Supplementary Material

Supplemental Material

## Figures and Tables

**Table:** 

	MA	AMA	HRS	MG	HAB	AMcA	KMAT
**Conceptualisation**			X			X	X
**Methodology**	X	X	X			X	X
**Formal Analysis**	X	X					
**Investigation**	X	X					
**Data Curation**	X	X	X	X			
**Writing – Original Draft**	X						
**Writing – Review and Editing**	X	X	X	X	X	X	X
**Funding acquisition**						X	X
