# Supplementary material for: Assessing the reach and engagement with the ‘How To Save A Life’ mass media campaign on drug-related death prevention in Scotland
Source: Drugs (Abingdon Engl). 2023 Sep 27;31(5):524–33. doi: 10.1080/09687637.2023.2262735 (PMC11441393; doi:10.1080/09687637.2023.2262735)

## **Appendix A – Key dates relating to the “How to save a life” campaign.**

| **Week beginning** | **Campaign dates** | **Radio** | **TV** | **Public transport** | **Outdoor/public displays** | **Social media** |
| --- | --- | --- | --- | --- | --- | --- |
| 30/08/2021 | Week of campaign launch |  |  |  |  |  |
| 06/09/2021 |  |  |  |  |  |  |
| 13/09/2021 |  |  |  |  |  |  |
| 20/09/2021 |  |  |  |  |  |  |
| 27/09/2021 |  |  |  |  |  |  |
| 04/10/2021 |  |  |  |  |  |  |
| 11/10/2021 |  |  |  |  |  |  |
| 18/10/2021 |  |  |  |  |  |  |
| 25/10/2021 |  |  |  |  |  |  |
| 01/11/2021 |  |  |  |  |  |  |
| 08/11/2021 |  |  |  |  |  |  |
| 15/11/2021 |  |  |  |  |  |  |
| 22/11/2021 |  |  |  |  |  |  |
| 29/11/2021 |  |  |  |  |  |  |
| 06/12/2021 |  |  |  |  |  |  |
| 13/12/2021 | December booster campaign |  |  |  |  |  |
| 20/12/2021 |  |  |  |  |  |  |
| 27/12/2021 |  |  |  |  |  |  |
| 03/01/2022 |  |  |  |  |  |  |
| 10/01/2022 |  |  |  |  |  |  |

## **Appendix B – ‘How to save a life’ mass media campaign materials.**

Poster 1


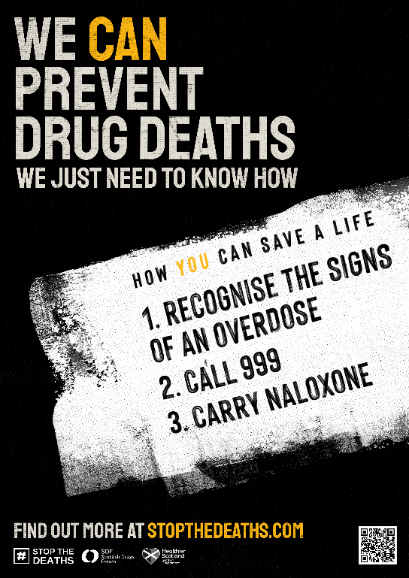


Poster 2


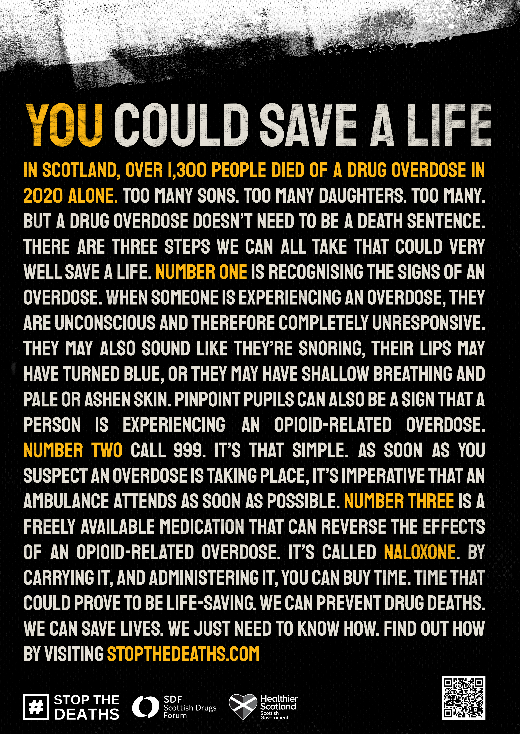


Poster 3


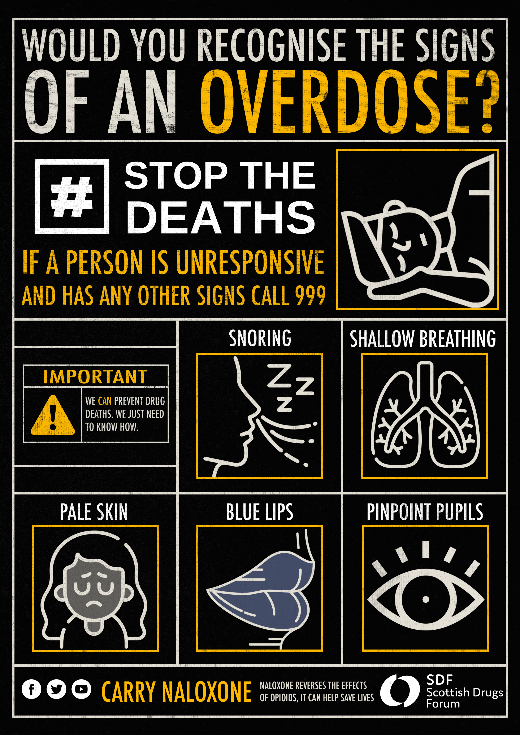


Social media


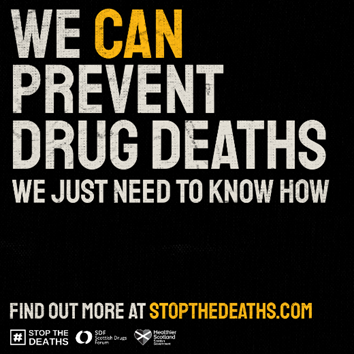


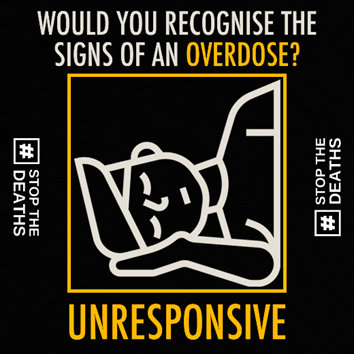


Taxi


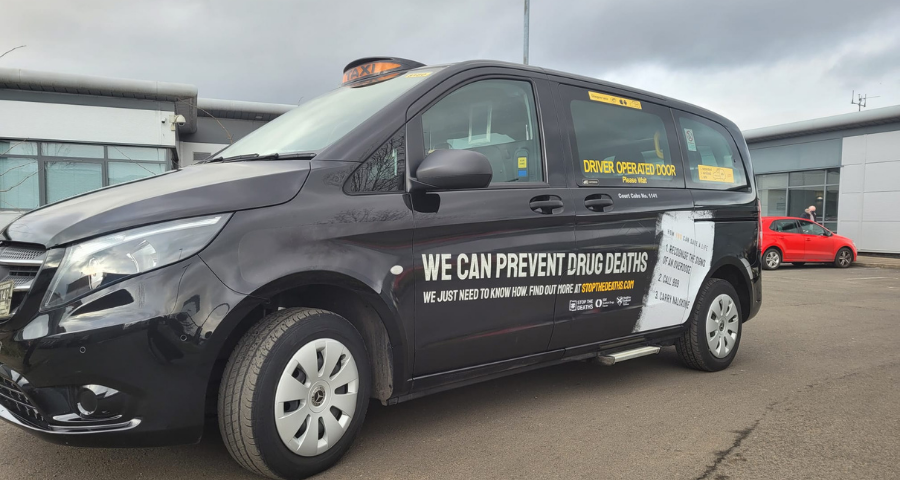

Supplement: Supplemental Material [file IDEP_A_2262735_SM0814.docx]
